# Co-expressing GroEL–GroES, Ssa1–Sis1 and Bip–PDI chaperones for enhanced intracellular production and partial-wall breaking improved stability of porcine growth hormone

**DOI:** 10.1186/s12934-020-01304-5

**Published:** 2020-02-18

**Authors:** Jinbo Deng, Jiaoqing Li, Miaopeng Ma, Peijing Zhao, Feiping Ming, Zhipeng Lu, Juqing Shi, Qin Fan, Qianyi Liang, Junhao Jia, Jiayi Li, Shuxia Zhang, Linghua Zhang

**Affiliations:** 1 Guangdong Provincial Key Laboratory of Protein Function and Regulation in Agricultural Organisms, Microbiological Staff Room, College of Life Sciences, South China Agricultural University, Wushan Road, Tianhe District, Guangzhou, 510642 Guangdong China; 2 Guangdong Laboratory for Lingnan Modern Agriculture, Guangzhou, Guangdong, 510642 China

**Keywords:** Porcine growth hormone, *Pichia pastoris*, Molecular chaperones, Intracellular soluble expression, Partial broken-wall

## Abstract

Porcine growth hormone (pGH) is a class of peptide hormones secreted from the pituitary gland, which can significantly improve growth and feed utilization of pigs. However, it is unstable and volatile in vitro. It needs to be encapsulated in liposomes when feeding livestock, whose high cost greatly limits its application in pig industry. Therefore we attempted to express pGH as intracellular soluble protein in *Pichia pastoris* and feed these yeasts with partial wall-breaking for swine, which could release directly pGH in intestine tract in case of being degraded in intestinal tract with low cost. In order to improve the intracellular soluble expression of pGH protein in *Pichia pastoris* and stability in vitro, we optimized the pGH gene, and screened molecular chaperones from *E. coli* and *Pichia pastoris* respectively for co-expressing with pGH. In addition, we had also explored conditions of mechanical crushing and fermentation. The results showed that the expression of intracellular soluble pGH protein was significantly increased after gene optimized and co-expressed with Ssa1–Sis1 chaperone from *Pichia pastoris.* Meanwhile, the optimal conditions of partial wall-breaking and fermentation of *Pichia pastoris* were confirmed, the data showed that the intracellular expression of the optimized pGH protein co-expressed with Ssa1–Sis1 could reach 340 mg/L with optimal conditions of partial wall-breaking and fermentation. Animal experiments verified that the optimized pGH protein co-expression with Ssa1–Sis1 had the best promoting effects on the growth of piglets. Our study demonstrated that Ssa1–Sis1 could enhance the intracellular soluble expression of pGH protein in *Pichia pastoris* and that partial wall-breaking of yeast could prevent pGH from degradation in vitro, release targetedly in the intestine and play its biological function effectively. Our study could provide a new idea to cut the cost effectively, establishing a theoretical basis for the clinic application of unstable substances in vitro.

## Introduction

The porcine growth hormone (pGH) is a single-chain polypeptide hormone produced by the secretion of the anterior pituitary gland [1–3]. pGH makes an important impact on the regulation of postnatal growth [2], effectively regulates metabolism [4] and promotes muscle growth [5].

The recombinant protein was secreted into the extracellular domain or located in the cytoplasm through the Golgi body after the endoplasmic reticulum synthesis process [6]. Proteins must be strictly checked before transported to ensure their correct folding [7]. The molecular chaperone is a class of molecules that play an important role in protein translation, transport, folding and modification [8]. *Pichia pastoris* Hsp40 family members have many ways to identify and delivery misfolded proteins [9]. Especially Ssa1 and Sis1, play a very important role in the formation of the correct native conformation of nascent peptides. Hsp 70 (including chaperones Bip and PDI) can identify the structural characteristics of most of the initial peptide chains to help them fold properly [10]. Bip can prevent the accumulation of errors in the peptide chain. PDI can identify disulfide bond to help the protein fold correctly, as supported by our previous observations [11]. And *E. coli* GroE (including chaperones GroEL and GroES) could form complexes with non-native polypeptides to avoid intermolecular aggregation formation of newly synthesized proteins [12]. Co-expression of molecular chaperones is a common method to enhance the intracellular soluble expression of recombinant proteins, which focused mainly on the *E. coli* expression system [13–15]. However, defects in prokaryotic posttranslational modification, as well as production of insoluble and non-secreted protein, limited the effectiveness of this system. Therefore, pGH expression studies have transferred to eukaryotic expression systems such as mammalian cell [16], yeast [17] and insect cell systems [18]. However, the yield of pGH in these eukaryotic expression systems is too low to be applied for production and the high cost is another limitation in its application.

It is well-known that *Pichia pastoris* protein expression system offers various advantages [19] including the success in high level production of a variety of heterologous proteins both intra- and extracellularly [20–22]. However, *Pichia pastoris* is a eukaryotic expression system, of which gene expression regulation system and material metabolic system were much more complex than prokaryotes expression systems, and the expression of some heterogeneous proteins in *Pichia pastoris* is unsatisfactory [11, 22]. Therefore, improvement of *Pichia pastoris* expression system is very important, such as the discovery of novel promoters, effective chaperones co-expression, optimization of transcriptional regulatory regions, optimization of target gene sequence, and selection of target gene copy number [23–25].

pGH could be successfully expressed in *Pichia pastoris* cells by optimizing the codon composition, and accounted for 10% of the intracellular total protein, which had the same activity as the growth hormone extracted from the pituitary gland [17], but this yield was still not high enough for commercialization. Meanwhile, pGH has a short half-life (20–30 min) and is easily degraded by protease like other proteins in the gastrointestinal tract [26]. pGH is not suitable for extracellular expression, therefore, we chose intracellular expression of pGH protein and hoped to expand the practical application of pGH in animal husbandry by using partially broken yeast cells for feeding. Until now, mechanical disruption had been used to completely break up cells and completely released intracellular substances [27], but not partial wall-breaking. Partial wall-breaking was beneficial to protect unstable proteins in cells and did not require liposomes for coating. At the same time, the time of partially broken cell walls degraded by intestinal enzymes was controllable. Therefore, targeted release of intracellular substances in intestine could be achieved by the use of partial wall-breaking process, which could promote to exert effectively the biological activity of intracellular proteins. Historically, pGH was derived from the pituitary glands of slaughtered pigs, limiting the available quantities and setting the price correspondingly high. By partially breaking the cell wall of yeast expressing pGH, the subsequent tedious purification steps were avoided, which brought about low-cost investment.

It was seldom reported how to cut down costs, promote effectively biological activity through partial wall-breaking and increase intracellular expression by molecular chaperone screening until now [28, 29]. The low yield of pGH was not suitable enough for commercialization. So we firstly optimized the nucleotide sequence of *pGH*. Secondly, in order to increase the intracellular yield, the optimized pGH was co-expressed with molecular chaperones from *Pichia pastoris* and *E. coli*, respectively. At the same time, the optimum parameters of partial wall-breaking and fermentation conditions were also investigated. Finally, the biological activity of pGH was further verified by cell and animal experiments. Our study laid a foundation for intracellular soluble expression of pGH protein in *Pichia pastoris* and provided a new direction for the application of substances easily degraded in vitro.

## Materials and methods

### Strains and plasmids

Strains *Pichia pastoris* X33, *E. coli* DH5α and plasmids pPICZA, pGro7, pPIC9K, pGAPZA, pGAPHA were purchased from Invitrogen (CA, USA). Plasmids pEASY-Blunt and pPAO [11] was kindly provided by the National Engineering Research Center of Plant Space Breeding (Guangzhou, China). Small intestinal epithelial cell line (IPEC-J2) was a gift from Deng Yiqun, South China Agricultural University. Landrace × Large White pigs were purchased from Swine Seed Breeding Center of Guangzhou and kept under standard farming conditions.

### Construction of expression plasmids

#### Cloning of pGH gene and construction of expression vector

To improve the expression level of recombinant pGH protein in *Pichia pastoris*, we used a novel deterministic computational algorithm COStar for codon optimization [30, 31]. The optimized *pGH* (GenBank: MH472824) has the following properties: enhanced *Pichia pastoris* codon usage bias of the heterologous gene, no unwanted cleavage sites for restriction enzymes and negative cis-acting elements, reduced number of highly repetitive nucleotide sequences, adaptive G + C content, and local formation of the transcribed mRNA secondary structure is inhibited. We designed a cloning approach based on synthetic overlapping primers and a PCR assembly strategy to construct a synthetic vector.

pGH fragment without signal peptide sequence (GenBank:X53325), the pGH cDNA (GenBank: M17704.1) which was obtained by amplification of the cDNA from the total RNA of porcine cerebellum by pGH primers [32], was used as template for optimization to generate a set of 12 overlapping oligonucleotides. A mixture of the oligonucleotides was used in first-round ten cycles of PCR procedure using KOD-FX polymerase to generate the coding sequence. Then, the region for Xho I/EcoRI restriction site was added to the 5′ terminal of the synthesized fragment in the second round PCR using the primer pGH-forward. At the same time, Xba I/NotI restriction site was linked to the 3′ terminal using the primer pGH-reverse [33]. The detailed steps were shown in Supplemental file. The complete artificial DNA was inserted into expression vector pPICZA to construct the plasmid pPICZA-optimized-pGH/pPICZA-pGH, which was confirmed by DNA sequencing.

#### Construction of chaperone co-expression plasmids

##### Construction of chaperone GroEL–GroES co-expression plasmids

Using the plasmid pGro7 as a template, primers GroEL-F/GroEL-R and primers GroES-F/GroES-R were used to amplify *GroEL* and *GroES* genes. Both *GroEL* and *GroES* genes were treated with Restriction endonuclease EcoRI and NotI respectively. After recovery, T4 ligase was used to connect the *GroEL* gene fragment or the *GroES* gene fragment with *Pichia pastoris* expression vector pGAPZA, which was also treated with both endonuclease EcoRI and NotI. Then the obtained plasmid pGAPZA–GroEL was treated with endonuclease BamHI mononuclease, and the plasmid pGAPZA–GroES was double digested with BamHI and BglII enzymes. The fragment of plasmid pGAPZA–GroEL was cut by the endonuclease BamHI mononuclease and the small fragment of plasmid pGAPZA–GroES was cut by the double enzymes BamHI and BglII. Fragments were connected by T4-link enzymes to construct pGAPZA–GroEL–GroES plasmid.

The GroEL–GroES expression box obtained after double-digesting the pGAPZA–GroEL–GroES plasmid with BglII and BamHI. Then the 9 K small fragment DNA containing the Amp coding frame and the pBR replication initiator obtained after digesting the pPIC9K plasmid by the endonuclease BglII enzyme were connected to construct the pGAPZA-LS-Amp plasmids.

The DNA fragment obtained by digesting pGAPZA-LS-Amp plasmid with BglII enzyme was dephosphorylated by CIAP and ligated with the 9 K large fragment DNA obtained by digesting the pPIC9K plasmid with BglII, to construct the pPIC9K-GAP-LS plasmid.

##### Construction of chaperone Ssa1–Sis1 co-expression plasmids

The hygromycin resistance gene ORF was amplified from pPAO plasmid, using the primer pair hyg-F/hyg-R. The resulting fragment was used to replace the Zeocin resistant gene located in pGAPZA plasmid by omega-PCR [30]. We designed a pair of chimeric primers for replacement of Zeocin resistant gene with hygromycin resistance gene. The 5′ sequences of the forward chimeric primer (Hyg-Zeo-F) and the reverse chimeric primer (Hyg-Zeo-R) were identical to the flanking sequences of Zeocin resistant gene, while the 3′ parts of these primers were respectively identical to the 5′ end and 3′ end of the hygromycin resistance gene. In the first PCR, the target hygromycin resistance gene was amplified from pPAO with chimeric primers. In the second PCR, the denatured strands of the PCR product containing the hygromycin resistance gene were served as megaprimers annealing to the flanking sequence of the Zeocin resistant gene in the plasmid pGAPZA. Thereby, the Zeocin resistance gene fragment was replaced by the target hygromycin resistance gene fragment in the de novo circular plasmid with two staggered nicks at the end (Additional file 1: Fig. S1). After treatment with DpnI, the PCR product was transformed into *E. coli* DH5α competent cells. The pGAPHA transformants were screened by a pair of primers, called hyg-F and hyg-R. We also constructed the G418-resistance plasmid, pGAPKA, by replacing the Zeocin resistance gene of the pGAPZA with the G418 resistance gene using the procedure described above (Additional file 1: Fig. S1).

The *Pichia pastoris Ssa1* gene was amplified from chromosomal DNA of *Pichia pastoris* X33 using the forward primer Ssa1-F and reverse primer Ssa1-R. The PCR product of *Ssa1* gene was digested with XhoI and XbaI and inserted into the vector pGAPKA resulting in pGAPKA–Ssa1 placing the gene under control of the GAP promoter. The *Pichia pastoris Sis1* gene was also amplified from chromosomal DNA of *Pichia pastoris* X33 using forward primer *Sis1*-F and reverse primer *Sis1*-R. The EcoRI/NotI amplicon was cloned into EcoRI/NotI-digested pGAPKA and pGAPHA resulting in pGAPKA-Ssa1 and pGAPHA-Sis1, respectively. In order to co-express both *Ssa1* and *Sis1*, the 5′ -untranslated region of GPR1 mRNA (RefSeq: NM_001180094) termed GPR, the internal ribosome entrysites (IRES) element from *S. cerevisiae* [31], was cloned into pGAPKA containing an upstream *Ssa1* and a downstream GRP. The GPR fragment was amplified from *S. cerevisiae S288c* genomic DNA with primers GPR-F/GPR-R.

Insertion omega-PCR [30] was used to construct pGAPKA–Ssa1–GPR (Additional file 1: Fig. S2). The target GPR being inserted into downstream of *Ssa1* in pGAPKA–Ssa1 was amplified from GPR fragment with chimeric primers GPR-Insert-F/GPR-Insert-R containing XhoI site in the first PCR. In the second PCR, the denatured strands of the GPR-containing PCR product were served as megaprimers annealing to the flanking sequences of the insertion site on the pGAPKA–Ssa1 plasmid to form a ῼ-shaped structure. Thereby, GPR fragment was integrated into the pGAPKA–Ssa1 (Additional file 1: Fig. S2). After treatment with DpnI, the PCR product was transformed into *E. coli* DH5α competent cells. The pGAPKA–Ssa1–GPR transformants were screened by a pair of primers GPR-F/GPR-R. The plasmid pGAPKA–Ssa1–GPR–Sis1 was constructed by inserting the *Sis1* gene into the XhoI–XbaI sites of plasmid pGAPKA-Ssa1-GPR.

##### Construction of chaperone Bip–PDI co-expression plasmids

Similar to the above procedure, insertion omega-PCR [30] was used to construct pGAPKA–PDI–GPR (Additional file 1: Fig. S3). The *Bip* and *PDI* genes were amplified from chromosomal DNA of *Pichia pastoris* X33 using primers Bip-F/Bip-R and PDI-F/PDI-R. The target GPR being inserted into downstream of *PDI* in pGAPKA–PDI was amplified from GPR fragment with chimeric primers GPR-Insert-F/GPR Insert-R containing XhoI site in the first PCR. In the second PCR, the denatured strands of the GPR-containing PCR product were served as megaprimers annealing to the flanking sequences of the insertion site on the pGAPKA–PDI plasmid to form a ῼ-shaped structure. Thereby, GPR fragment was integrated into the pGAPKA–PDI (Additional file 1: Fig. S3). After treatment with DpnI, the PCR product was transformed into *E. coli* DH5α competent cells. The pGAPKA–PDI–GPR transformants were screened by a pair of primers GPR-F/GPR-R. The plasmid pGAPKA–Bip–GPR–PDI was constructed by inserting the *Bip* gene into the XhoI–XbaI sites of plasmid pGAPKA–PDI–GPR. All primers were listed in Additional file 1: Table S1. All plasmids were confirmed by DNA sequencing, and the plasmids used in this study were shown in Additional file 1: Table S2 and Fig. S4.

### Generation of *Pichia pastoris* strain expressing intracellular recombinant optimized pGH

*Pichia pastoris* X33 electro-competent cells were transformed with SacI-linearized pPICZA-pGH. Positive transformants were screened on methanol (BMGY) containing 1000 μg/mL Zeocin. And X33 colonies were further verified by colonies PCR using universal primers 5′ AOX1 and 3′ AOX1.

*Pichia pastoris* X33 electro-competent cells were transformed with SacI-linearized pPICZA-optimized-pGH. Positive transformants were screened on BMGY containing 1000 μg/mL Zeocin. Transformants confirmed by PCR were grown overnight in BMGY to prepare for the competent cells, and they will be used for the second round of electroporation. For the construction of strains co-expressing with chaperones. BlnI-linearized pPIC9K-GAP-LS, pGAPKA–Ssa1–GPR–Sis1 and pGAPKA–Bip–GPR–PDI were transformed into strains harboring different copy of optimized pGH gene by electroporation respectively. The transformants were screened on BMGY plates containing 1000 μg/mL G418.

### Small-scale pGH expression and purification

Transforming strains were cultivated in BMGY culture medium containing 100 mM potassium phosphate (pH 6.0), 1.34% (w/v) yeast nitrogen base without amino acids, 4 × 10^−5^% (w/v) biotin, and 0.5% glycerol (w/v) (BMGY), respectively. Cultivations were performed in 500 mL flasks containing 50 mL of BMGY and were incubated at 20 °C with 230 rpm for 12 h, 24 h, 36 h and 48 h respectively. Then, the cells were centrifuged at 3500 rpm for 5 min, and the cell pellet was resuspended in 50 mL of BMGY. To maintain the expression of the product, 100% of methanol was added to a final concentration of 0.5% (v/v) every 24 h. After 48 h of induction, cells were collected by centrifugation. The samples were resuspended in 300 μL of Lysis buffer. An equal volume of acid-washed glass beads was added, and cells were disrupted by vortexing ten times for 30 s at 4 °C. The lysate was centrifuged at 5000 rpm for 10 min at 4 °C and supernatant was collected.

The supernatant of soluble pGH was applied onto a HisTrap affinity chromatography column (BIO-RAD, Poly-Prep Chromatography Columns, Catalog 731-1550), which was previously equilibrated with 50 mM Tris–HCl buffer (pH 7.5). The column was washed with 6 × bed volumes of the same buffer containing 5 mM imidazole to obtain F liquid. Then the column was washed with 5 mL 10 mM imidazole in the same buffer to obtain W1 fraction, and repeated this step to obtain W2 fraction. 0.5 mL 250 mM imidazole in the same buffer was added to column to obtain E1 fraction, and then repeat three times to obtain E2, E3 and E4 fractions.

Similarly, the supernatant of insoluble pGH was applied onto a HisTrap affinity chromatography column. The column was washed with 6 × bed volumes of the same buffer containing 8 M urea (pH 8.0) to obtain F liquid. Then the column was washed with 5 mL 8 M urea (pH 6.3) in the same buffer to obtain W1 fraction, and repeated this step to obtain W2 fraction. 0.5 mL 8 M urea (pH 4.5) in the same buffer was added to column to obtain E1 fraction, and then repeat three times to obtain E2, E3 and E4 fractions. The flow rate was 1.0 mL/min, and all fractions were subjected to SDS–polyacrylamide gel electrophoresis.

### Extraction of intracellular proteins from *Pichia pastoris*

Protein extractions from cytoplasmic and membrane associated fractions were done according to Shen et al. [34]. Briefly, strains were harvested and centrifuged at 5000 rpm for 5 min when the OD_600_ reached 6, and the strains were washed in PBS (pH 7.4), then resuspended in 300 μL of Lysis buffer. An equal volume of acid-washed glass beads was added, and cells were disrupted by vortexing ten times for 30 s at 4 °C The lysate was centrifuged at 5000 rpm for 10 min at 4 °C and supernatant was collected. The pellet was further resuspended in 100 μL lysis buffer plus 2% SDS. After centrifugation at 5000 rpm for 5 min at 4 °C, the supernatants containing the membrane-associated proteins were collected. 20 μg of cytoplasmic proteins or membrane associated proteins was analyzed by SDS–PAGE and Western blot whose concentration was determined by BCA protein assay (Additional file 1: Fig. S5).

### SDS–PAGE analysis and Western blot assays

Equal amounts of extracted intracellular proteins were analyzed on 15% (w/v) SDS polyacrylamide gel electrophoresis (PAGE), after electrophoresis, gels were stained with Coomassie blue R-250 for 2 h. Then 0.25 M KCl decolorizing solution was added and decolorized for many times until the protein band was clearly visible. For Western blot assay, the proteins were transferred to PVDF membrane and the membrane was incubated at 37 °C with 50 mL 5% (w/v) skimmed milk for 2 h. And then the membrane was washed 3 times with PBS for 15 min each time. Afterward, the membrane was incubated with a 1:3000 dilution of pGH polyclonal antibody [33] overnight at 4 °C and incubated with HRP conjugated goat anti-rabbit IgG (CWBIO, China) at a dilution of 1:5000 at 37 °C for 1 h. After each incubation, the membrane was washed 3 times with PBST for 15 min each time. Immunoreactive bands were visualized with Enhanced HRPDAB Chromogenic Substrate Kit (TIANGEN, China). Protein bands on SDS–PAGE were estimated by ImageJ software.

### Optimization of fermentation conditions of pGH in *Pichia pastoris*

The pGH1-Ssa1/Sis1 strains were scraped from the BMGY plate and placed in 250 mL shake flasks containing 50 mL BMGY with 0.5% (v/v) methanol at 28 °C, pH 6.0, and 200 rpm for 16-24 h. The seed liquids were transferred to shake flasks and fermented under conditions (temperature (15 °C, 20 °C, 25 °C and 30 °C) and pH (3.0. 4.0, 5.0, 6.0 and 7.0) for 48 h, respectively). After crushing, intracellular expression levels of pGH protein were detected by SDS–PAGE in the whole cell samples and the supernatant samples of *Pichia pastoris* cells.

### pGH protein biological activity analysis

pGH protein biological activity analysis was evaluated by MTT method [35]. Briefly, IPEC-J2 cell line, a gift from Deng Yiqun, South China Agricultural University, were maintained in Dulbecco’s MEM nutrient mix F12 (DMEM-F12) (1:1) (Gibico, Guangzhou, China) with 10% (v/v) FCS (Gibico, Guangzhou, China) at 37 °C in 5% CO_2_. Prior to the treatment, IPEC-J2 cells were resuspended by trypsinization with trypsin–EDTA (0.25%) and seeded into 24-well plates at 2 × 10^5^ cells/well without antibiotics (final concentration of the cell suspension was 5 × 10^4^ cells/mL). The cells were inoculated to 96 wells and cultured for 18 h at 5% CO_2_, 37 °C. After cell adherence, pGH was added to the final concentrations of 0, 25, 75, 125 μg/L and cultured for 24 h, 36 h, 48 h for MTT assay and cell number count (cell viability after treatment exceeded 90% as assessed by Trypan blue dye exclusion), respectively. Then 10 μL MTT solution was added to each well for 4 h. Finally, 150 μL dimethyl sulfoxide was added to each well and placed on a shaking at 200 rpm for 15 min, so that the crystallinity was fully dissolved and the absorbance value at OD_490nm_ was measured (Stimulation Index (%) = The OD value of the experimental group/The OD value of the control group × 100%).

### Preparation of *Pichia pastoris* X33 with partial broken wall

Based on the results from “SDS–PAGE analysis and western blot assays” section, effect of Ssa1–Sis1 was most remarkable among the three conditions. Therefore, optimized-pGH-Ssa1/Sis1 (pGH1–Ssa1/Sis1) *Pichia pastori* X33 was used for further investigation. After pGH expression, pGH1–Ssa1/Sis1 *Pichia pastori* X33 was crushed by JN-02C Low Temperature Ultra High Pressure Continuous Flow Cell Breaker (JNBIO, Guangzhou, China) under pressure of 300, 500, 700, 900, 1100, 1300 and 1500 bar, respectively. According to the Chinese Pharmacopoeia, simulated gastric fluid (as follows: 1.64 mL diluted hydrochloric acid and 1 g pepsin were dissolved in 100 mL pure water) and simulated intestinal fluid (as follows: 1 g trypsin and 0.68 g KH_2_PO_4_ were dissolved in 100 mL pure water, the pH was adjusted to 6.8 by 0.1 M NaOH) were prepared [36]. To determine the content of pGH1-Ssa1/Sis1 protein in *Pichia pastoris* X33, 1 g pGH1–Ssa1/Sis1 *Pichia pastori* X33 was collected by centrifugation after adding to 100 mL simulated gastric fluid for 6 h [37], then added to 100 mL simulated intestinal fluid and the amount of pGH1–Ssa1/Sis1 protein was determined by ELISA every hour [38]. Briefly, samples after incubation with the simulated intestinal fluid were incubated with a 1:3000 dilution of pGH polyclonal antibody [33] overnight at 4 °C and incubated with HRP conjugated goat anti-rabbit IgG (CWBIO, China) at a dilution of 1:5000. The reaction was visualized with TMB and stopped with 1 M H_2_SO_4_ and resulted in an OD_450_ value. Results were representative of three biological replicates.

### Clinical observation

Landrace × Large White pigs were used in this study. Ten 22-day-old piglets were divided into five groups. The A group was orally administrated with PBS, the B group was orally administrated with 2 mg/kg body weight purified pGH, the C group was orally administrated with 10 mg/kg body weight *Pichia pastoris* X33/pGH1 with non-broken wall, the D group was orally administrated with 10 mg/kg body weight *Pichia pastoris* X33/pGH1 with partial-broken wall, the E group was orally administrated with 10 mg/kg body weight *Pichia pastoris* X33/pGH1–Ssa1/Sis1 with partial broken wall. The dosage of 2 mg/kg body weight purified pGH (10 mg *Pichia pastoris* X33/pGH1–Ssa1/Sis1 with partial broken wall was equivalent to 2 mg purified pGH based on results from “pGH protein biological activity analysis” section was used for oral administration since we have previously found this to yield better growth-promoting responses in piglets in our experiments (Zhang et al., unpublished data). All formulations were delivered in a total volume of 5 mL and were applied using a 1 cm 3 tuberculin syringe attached to an 18-gauge feeding needle, which were passed down the oral cavity and the esophagus, and into the stomach. The treatment for each group was repeated once with a 3-day interval post first administration. The body weight of piglets was measured and the growth rate was calculated every 14 days. The growth rate was calculated according to the statistics of weight growth/growth time. All animal experiments were performed according to national regulations and institutional guide-lines.

### Statistical analysis

Data were analyzed using the Statistical Analysis System (SAS 9.1.3). Differences in expression levels were investigated using one-way analysis of variance (ANOVA). Means of the values were compared using Duncan’s multiple comparison tests. A *p* value of < 0.05 was considered significant.

## Results

### Construction of recombinant strains of pGH gene and expression of pGH proteins

**Fig. 1:**
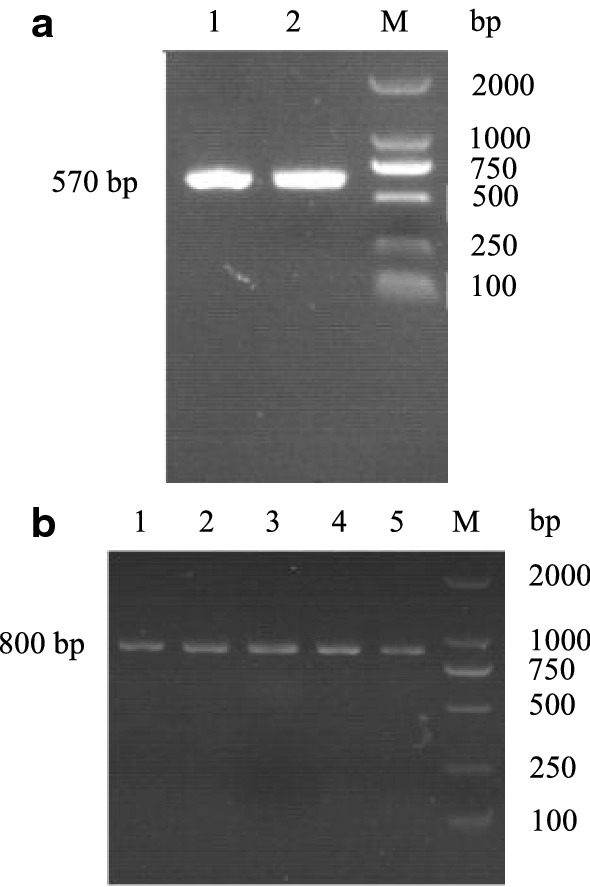
Cloning and recombination of pGH. **a** Cloning of *pGH* genes (GenBank: X53325). Using the cDNA of *pGH* gene obtained after the reverse transcription of total cerebellum RNA as a template, the *pGH* full-length gene was cloned by amplification of *pGH* gene primer. Lane 1, EcoRI and NotI amplification product with enzyme cleavage site, lane 2, XhoI and XbaI amplification product with enzyme cleavage site, lane M, DL2000 standard molecular weight DNA Marker. **b** Construction recombination of pPICZA–pGH. pPICZA–pGH was linearized by SacI, to transformed into *Pichia pastoris* X33, which was screened by Zeocin, and the colony PCR was performed with general primers 5′ AOX1 and 3′ AOX1. Lanes 1–5, the intracellular expression of pGH in *Pichia pastoris* X33, lane M, DL2000 standard molecular weight DNA Marker

**Fig. 2:**
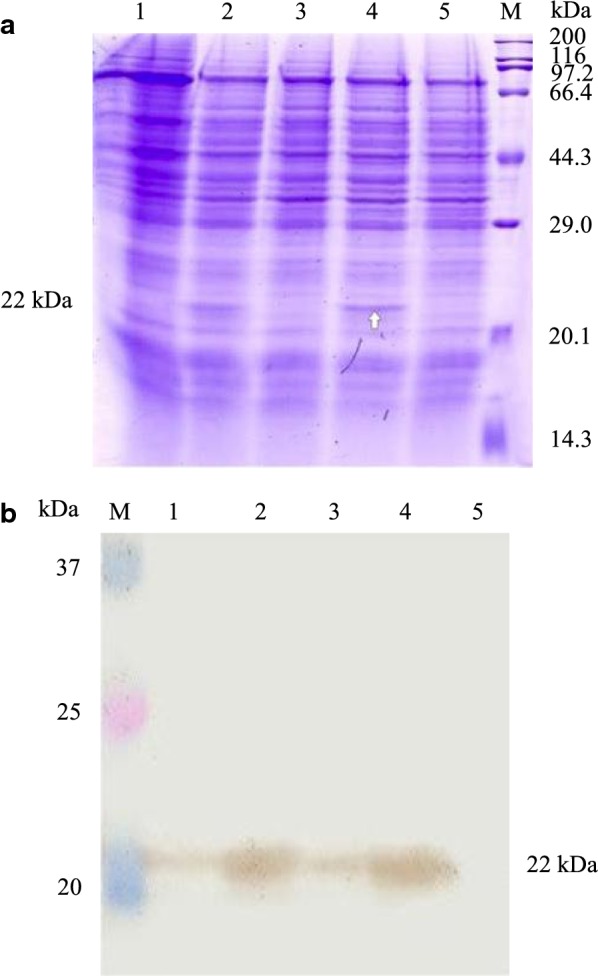
Expression of pGH in *Pichia pastoris* X33. **a** Intracellular expression of pGH protein by SDS–PAGE. Lane 1, *Pichia pastoris* X33 of control sample, lane 2, the expression of pGH protein in the whole cells after cultivation for 12 h, lane 3, the expression of pGH protein in the broken cells supernatant after cultivation for 12 h, lane 4, the expression of pGH protein in the whole cells after cultivation for 24 h, lane 5, the expression of pGH protein in the broken cells supernatant after cultivation for 24 h, lane M, the standard molecular weight Marker of 3452 wide type protein. **b** Intracellular expression of pGH protein by Western-blot. Lane M, Dual Color standard molecular weight pre-dye Marker, lane 1, the expression of pGH protein in the broken cells supernatant after cultivation for 12 h, lane 2, the expression of pGH protein in the whole cells after cultivation for 12 h, lane 3, the expression of pGH protein in the broken cells supernatant after cultivation for 24 h, lane 4, the expression of pGH protein in the whole cells after cultivation for 24 h, lane 5, *Pichia pastoris* X33 of control sample

By extracting the total RNA from porcine cerebellum and obtaining cDNA from reverse transcriptase, the *pGH* gene was obtained by pGH primer amplification. The amplification products and pPICZA vectors were connected by EcoRI and NotI double enzyme digestion, and the intracellular expression vector pPICZA–pGH of *Pichia pastoris* was constructed. Then pGH single and muticopy strains were generated by transformation of *Pichia pastoris* X33 with the linearized vector pPICZA–pGH. After PCR amplification, a stripe with the same size (pGH specific uniform bands appeared at 570 bp) as the target gene was shown in Fig. 1. Meanwhile, SDS–PAGE and Western-blot were used to detect the intracellular protein secretion of pGH in *Pichia pastoris* cells. We observed the specific protein bands in the whole cell lanes with 22 kDa (Fig. 2), while the same protein bands were not visible in the corresponding cell broken supernatant.

Therefore, *Pichia pastoris* expression vector with *pGH* gene was successfully constructed, and pGH protein expressed in *Pichia pastoris* cells was mainly in the form of inclusion bodies.

### Intracellular expression of pGH protein in *Pichia pastoris* X33 after optimization of pGH gene

**Fig. 3:**
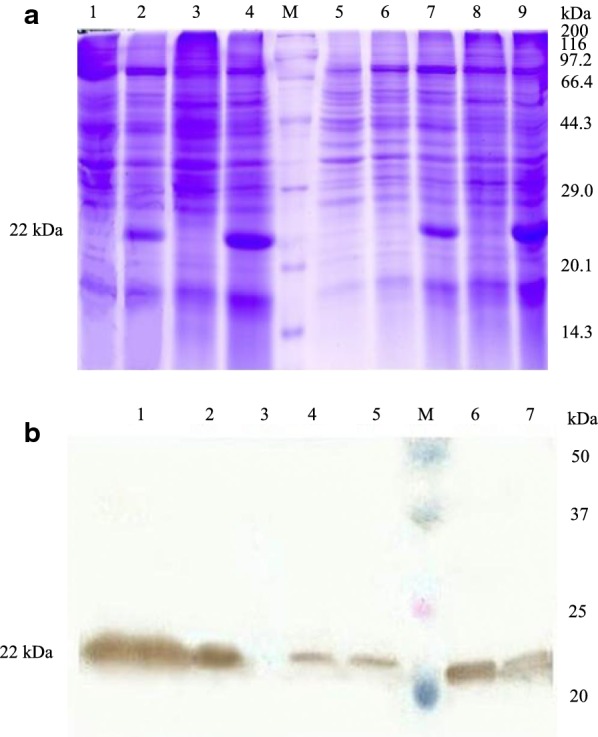
Intracellular expression of pGH in *Pichia pastoris* X33 after gene optimization. **a** SDS–PAGE analysis of soluble and insoluble protein fractions of *Pichia pastoris* X33 expressing optimized *pGH* under the control of AOX1 promoter after induction at 30 °C. Lanes 1 and 6, non optimized pGH gene for the expression of soluble pGH protein in broken cells supernatant after induction cultivation for 12 h and 24 h, lanes 2 and 7, non optimized *pGH* gene for the expression of insoluble pGH protein in whole cells after induction cultivation for 12 h and 24 h, lanes 3 and 8, optimized *pGH* gene for the expression of soluble pGH protein in broken cells supernatant after induction cultivation for 12 h and 24 h, lanes 4 and 9, optimized *pGH* gene for the expression of insoluble pGH protein in whole cells after induction cultivation for 12 h and 24 h, lane M, the standard molecular weight Marker of 3452 wide type protein, lane 5, *Pichia pastoris* X33 of control sample. **b** Western-blot analysis of pGH protein of *Pichia pastoris* X33 expressing optimized *pGH* under the control of AOX1 promoter after induction at 30 °C for 24 h. lane 1, optimized *pGH* gene for the expression of insoluble pGH protein in broken cells sedimentation, lane 2, non optimized *pGH* gene for the expression of insoluble pGH protein in broken cells sedimentation, lane 3, *Pichia pastoris* X33 of control sample, lane 4, optimized *pGH* gene for the expression of soluble pGH protein in broken cells supernatant, lane 5, non optimized *pGH* gene for the expression of soluble pGH protein in broken cells supernatant, lane M, Dual Color standard molecular weight pre dye Marker, lane 6, optimized *pGH* gene for the expression of total pGH protein in whole cells, lane 7, non optimized *pGH* gene for the expression of total pGH protein in whole cells

COStar software was used to optimize the encoding sequence of *pGH*. The optimized results were that the base sequence similarity between the optimized sequence and the original sequence was 70.35%, and the GC value in the optimized sequence was 43.0%. The full length of the optimized *pGH* gene was obtained by overlapping PCR (Additional file 1: Figs. S6 and S7). Then the recombinant strains containing optimized *pGH* gene were generated by transformation of *Pichia pastoris* X33 with the linearized intracellular vector pPICZA. The clone X33-pPICZA-optimized pGH (pGH1) strain was induced with 0.5% (v/v) methanol for gene expression at 30 °C for 24 h, the cells were collected by centrifugation, then the pGH protein yield was detected by SDS–PAGE and Western-blot. Data showed that compared with the control group, the optimized pGH had a strong up-regulation effect on total pGH protein of *Pichia pastoris* cells after 24 h of induction (Fig. 3a). However, the majority of the expressed pGH is associated to the inclusion bodies as insoluble protein (Fig. 3), and the yield of soluble pGH protein in the cells was not significantly enhanced as judged from the intensity of protein bands.

### Construction of recombinant strains containing optimized pGH gene and chaperone genes

#### Co-expression of *E. coli* chaperone GroEL–GroES protein and pGH protein in *Pichia pastoris*

**Fig. 4:**
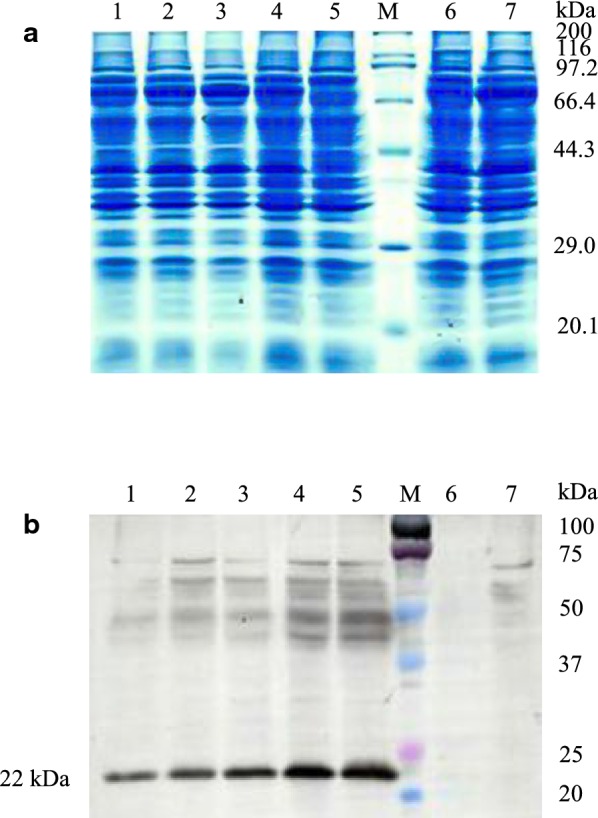
Detection of co-expression of pGH with GroEL–GroES in *Pichia pastoris.***a** SDS–PAGE analysis of soluble protein fractions from *Pichia pastoris* X33 expressing optimized *pGH* alone and with GroEL–GroES co-expression. Lanes 1–5, samples of the intracellular supernatant of the co-expressing strain after induction, lane M, the standard molecular weight Marker of 3452 wide type protein, lane 6, the sample of the intracellular supernatant of the co-expressing strain before induction, lane 7, *Pichia pastoris* X33 of control sample. **b** Western-blot analysis of soluble protein fractions from *Pichia pastoris* X33 expressing optimized *pGH* alone and with GroEL–GroES co-expression. Lanes 1–5, samples of the intracellular supernatant of the co-expressing strain after induction, lane M, the standard molecular weight Marker of 3452 wide type protein, lane 6, the sample of the intracellular supernatant of the co-expressing strain before induction, lane 7, *Pichia pastoris* X33 of control sample. The G418 concentration of the expression strains of lanes 1–3 was 1.0 mg/mL, the G418 concentration of the expression strains of lanes 4 and 5 was 2.0 mg/mL

The pGH1–GroEL/GroES strain was obtained by genetic engineering. The strains were cultured in BMGY medium. The expression of pGH protein in the broken samples supernatant was detected. The results showed that after co-expression of GroEL–GroES molecular chaperones with optimized pGH gene at 30 °C for 24 h, there were no visible pGH protein band in the cells’ supernatant. The results of Western-blot showed that co-expression of molecular chaperones GroEL–GroES could increase expression level of soluble pGH protein (Fig. 4).

#### Co-expression of Pichia pastoris chaperone Ssa1–Sis1 protein and pGH protein in Pichia pastoris

**Fig. 5:**
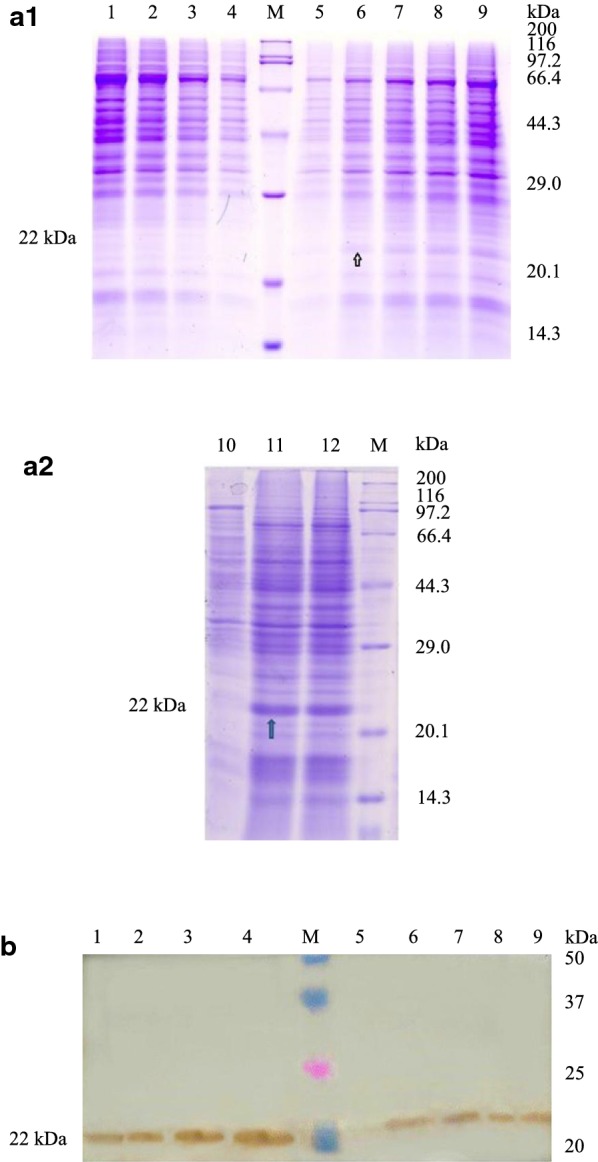
Detection of co-expression of pGH with Ssa1–Sis1 in *Pichia pastoris.***a** SDS–PAGE analysis of pGH protein from *Pichia pastoris* X33 expressing optimized *pGH* alone and with Ssa1–Sis1 co-expression. Lanes 1–4, X33-pPICZA-optimized pGH (pGH1) for the expression of soluble pGH protein in broken cells supernatant cultivation for 48 h, 36 h, 24 h, and 12 h, lane 5, *Pichia pastoris* X33 of control sample, lane M, the standard molecular weight Marker of 3452 wide type protein, lanes 6–9, X33-pPICZA-optimized pGH–Ssa1–Sis1 (pGH1–Ssa1/Sis1) for the expression of soluble pGH protein in broken cells supernatant cultivation for 12 h, 24 h, 36 h, and 48 h, lane 10, *Pichia pastoris* X33 of control sample, lanes 11 and 12, pGH1 and pGH1–Ssa1/Sis1 for the expression of total pGH protein in whole cells cultivation for 48 h. **b** Western-blot analysis of intracellular soluble pGH protein fractions from *Pichia pastoris* X33 expressing optimized *pGH* alone and with Ssa1–Sis1 co-expression. Lanes 1–4, X33-pPICZA-optimized pGH–Ssa1–Sis1 (pGH1–Ssa1/Sis1) for the expression of soluble pGH protein in broken cells supernatant cultivation for 12 h, 24 h, 36 h, and 48 h, lane M, Dual Color standard molecular weight pre dye Marker, lane 5, *Pichia pastoris* X33 of control sample, lanes 6–9, X33-pPICZA-optimized pGH (pGH1) for the expression of soluble pGH protein in broken cells supernatant cultivation for 12 h, 24 h, 36 h, and 48 h

The pGH1-Ssa1/Sis1 strain was obtained by genetic engineering. The strain was cultured in BMGY medium containing 0.5% (v/v) methanol, then the strains were collected by centrifugation every 12 h, to analyze the content of pGH protein both in *Pichia pastoris* total cell protein and intracellular soluble protein. The results showed that after centrifugation of extracts of these strains of *Pichia pastoris*, the pGH target protein-specific bands (soluble pGH protein) can be detected in the supernatant of broken pGH1–Ssa1/Sis1 cells (Fig. 5a). However, the total yield of pGH protein expression in *Pichia pastoris* cells was not increased in our study. Western-blot detection results also showed that co-expression of yeast chaperones Ssa1–Sis1 with the optimized pGH dramatically improved the intracellular soluble pGH production in *Pichia pastoris* (Fig. 5b).

#### Co-expression of *Pichia pastoris* chaperone Bip–PDI protein and pGH protein in Pichia pastoris

**Fig. 6:**
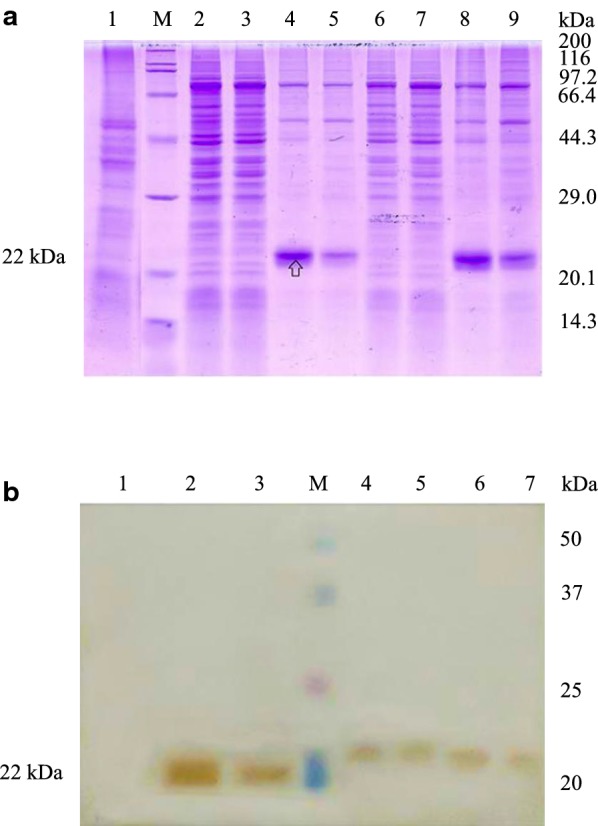
Detection of co-expression of pGH with Bip–PDI in *Pichia pastoris.***a** SDS–PAGE analysis of soluble and insoluble protein fractions from *Pichia pastoris* X33 expressing optimized *pGH* alone and with Bip–PDI co-expression. Lane 1, *Pichia pastoris* X33 of control sample, lane M, the standard molecular weight Marker of 3452 wide type protein, lanes 2 and 3, X33-pPICZA-optimized pGH (pGH1) and X33-pPICZA-optimized pGH–Bip–PDI (pGH1–Bip/PDI) for the expression of soluble pGH protein in broken cells supernatant for 12 h, lanes 4 and 5, pGH1 and pGH1–Bip/PDI for the expression of insoluble pGH protein in broken cells sedimentation for 12 h, lanes 6 and 7, pGH1 and pGH1-Bip/PDI for the expression of soluble pGH protein in broken cells supernatant for 24 h, lanes 8 and 9, pGH1 and pGH1–Bip/PDI for the expression of insoluble pGH protein in broken cells sedimentation for 24 h. **b** Western-blot analysis of soluble and insoluble protein fractions from *Pichia pastoris* X33 expressing optimized *pGH* alone and with Bip–PDI co-expression. Lane 1, *Pichia pastoris* X33 of control sample, lanes 2 and 3, X33-pPICZA-optimized pGH (pGH1) and X33-pPICZA-optimized pGH–Bip–PDI (pGH1–Bip/PDI) for the expression of total pGH protein in whole cells cultivation for 12 h, lane M, Dual Color standard molecular weight pre dye Marker, lanes 4 and 5, pGH1 and pGH1–Bip/PDI for the expression of soluble pGH protein in broken cells supernatant cultivation for 12 h, lanes 6 and 7, pGH1 and pGH1–Bip/PDI for the expression of soluble pGH protein in broken cells supernatant cultivation for 24 h

The pGH1-Bip/PDI strain was obtained by genetic engineering. The strains were cultured in BMGY medium containing 0.5% (v/v) methanol, then the cells were collected by centrifugation every 12 h. The results showed that after co-expression of Bip–PDI molecular chaperones with optimized *pGH* gene at 30 °C for 24 h, there were no visible pGH protein band in the cells’ supernatant (SDS–PAGE), and the yield of insoluble pGH protein showed a decrease after induction in *Pichia pastoris* cells (Fig. 6). The results of Western-blot showed that co-expression of molecular chaperone Bip–PDI reduced the expression level of the pGH protein, meanwhile the amount of soluble pGH protein in the supernatant remained unchanged (Fig. 6b).

### Effect of cultivation conditions on intracellular soluble pGH protein yield in pGH1-Ssa1/Sis1

#### Effect of temperature in the expression of pGH1–Ssa1/Sis1 protein in *Pichia pastoris*

**Fig. 7:**
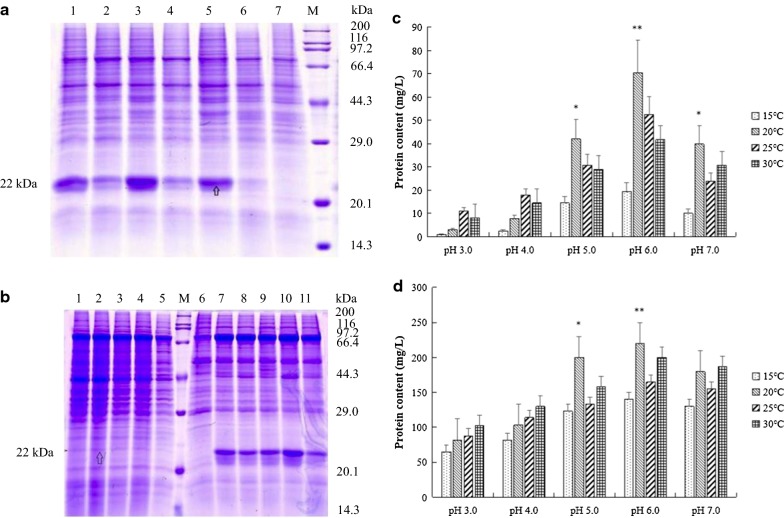
Effect of cultivation conditions on intracellular soluble pGH protein yield in X33-pPICZA-optimized pGH1–Ssa1–Sis1 (pGH1–Ssa1/Sis1). **a** Effect of temperature on expression of protein in pGH1-Ssa1/Sis1 of *Pichia pastoris*. Lane 1, expression of total pGH protein in the whole cells cultivation at 15 °C, lane 2, expression of soluble pGH protein in broken cells supernatant at 15 °C, lane 3, expression of total pGH protein in the whole cells cultivation at 20 °C, lane 4, expression of soluble pGH protein in broken cells supernatant at 20 °C, lane 5, expression of total pGH protein in the whole cells cultivation at 25 °C, lane 6, expression of soluble pGH protein in broken cells supernatant at 25 °C, lane 7, *Pichia pastoris* X33 of control sample, lane M, the standard molecular weight Marker of 3452 wide type protein. **b** Effect of pH on expression of protein in X33-pPICZA-optimized pGH1–Ssa1–Sis1 (pGH1–Ssa1/Sis1) of *Pichia pastoris*. Lanes 1–5, expression of soluble pGH protein in broken cells supernatant with pH values of 3.0, 4.0, 5.0, 6.0 and 7.0, lane M, the standard molecular weight Marker of 3452 wide type protein, lane 6, *Pichia pastoris* X33 of control sample, lanes 7–11, expression of total pGH protein in the whole cell samples with pH values of 3.0, 4.0, 5.0, 6.0 and 7.0. **c** Soluble level of pGH protein (mg protein per liter of fermentation broth) after fermentation in optimized-pGH–Ssa1/Sis1 (pGH1–Ssa1/Sis1) of *Pichia pastori* X33. **d** Insoluble level of pGH protein (mg protein per liter of fermentation broth) after fermentation in optimized-pGH–Ssa1/Sis1 (pGH1–Ssa1/Sis1) of *Pichia pastoris* X33. The results of three independent experiments were indicated as mean ± SD. *p < 0.05 indicated significant differences vs. 15 °C group, **p < 0.01 indicated highly significant differences vs. 15 °C group

In our study, the yield of soluble pGH protein with co-expressed Ssa1/Sis1 was the highest. So pGH1–Ssa1/Sis1 was used to research the effect of culture conditions on intracellular soluble pGH protein amounts in *Pichia pastoris*. The pGH1–Ssa1/Sis1 strains were inoculated on BMGY containing 0.5% (v/v) methanol, and the bacteria were incubated at 25 °C, 20 °C and 15 °C, respectively. After crushing, the pGH protein expression in whole cells and in the intracellular soluble supernatant by SDS–PAGE (Fig. 7a). SDS–PAGE results showed that pGH1–Ssa1/Sis1 strains could express pGH protein in *Pichia pastoris* cells in different levels at different temperatures. The expression level of pGH was low at 25 °C and 15 °C. The yield of pGH was the highest at 20 °C, and the production of intracellular soluble pGH protein in the supernatant was also the highest at 20 °C.

#### Effect of pH in the expression of pGH1–Ssa1/Sis1 protein in *Pichia pastoris*

The pGH1–Ssa1/Sis1 strains were inoculated on BMGY containing 0.5% (v/v) methanol, and the strains were incubated at pH 3.0, 4.0, 5.0, 6.0 and 7.0, respectively. After crushing, the whole cell samples and the supernatant samples were detected by SDS–PAGE for intracellular expression of pGH protein in *Pichia pastoris* cells. SDS–PAGE results showed that in *Pichia pastoris* pGH1–Ssa1/Sis1, the expression of pGH protein in the cells’ supernatant was not obviously changed at different pH values, and the yield of pGH protein in the whole cell samples at pH 6.0 was the highest (Fig. 7b).

From all above results we could concluded that the best culture condition for pGH1–Ssa1/Sis1 strains to express pGH protein in *Pichia pastoris* cells is at 20 °C and pH 6.0, for 48 h.

### Purification of pGH protein after expression in *Pichia pastoris*

**Fig. 8:**
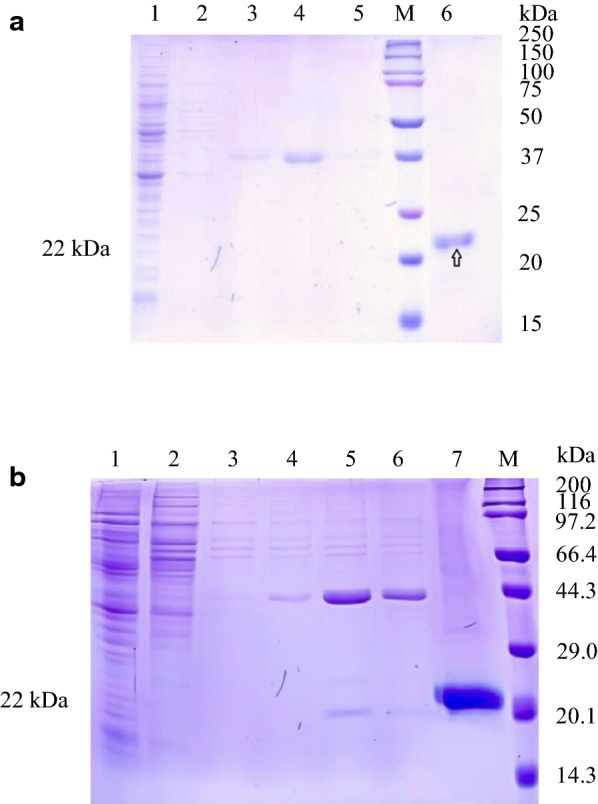
Purification of pGH protein after fermentation in optimized-pGH–Ssa1/Sis1 (pGH1–Ssa1/Sis1) of *Pichia pastori* X33. **a** Intracellular soluble pGH protein in broken cells supernatant, lane 1, F fraction, lane 2, W1 fraction, lane 3, W2 fraction, lane 4, E1 fraction, lane 5, E2 fraction, lane M, Dual Color standard molecular weight pretreated Marker, lane 6, E3 fraction. **b** The insoluble pGH protein in broken cells sedimentation. Lane 1, F fraction, lane 2, W1 fraction, lane 3, W2 fraction, lane 4, E1 fraction, lane 5, E2 fraction, lane 6, E3 fraction, lane 7, E4 fraction, lane M, the standard molecular weight Marker of 3452 wide type protein

The pGH1–Ssa1/Sis1 strains were inoculated in BMGY containing 0.5% (v/v) methanol, the strains were collected at 20 °C with pH 6.0, for 48 h. After crushing, the samples were recovered and purified by nickel affinity chromatography. The SDS–PAGE results showed that both soluble pGH protein and insoluble pGH protein were purified by nickel affinity chromatography (Fig. 8a, b), and there were specific protein bands around 22 kDa. After purification, the concentration of pGH protein was determined by BCA protein quantitative method. Compared to controls, the results showed (Fig. 7c, d) yields for the intracellular soluble pGH protein of 70 mg/L (*p *< 0.01), insoluble pGH protein of 270 mg/L, and total pGH protein of in cells was 340 mg/L (*p *< 0.01).

### pGH protein biological activity analysis

**Fig. 9:**
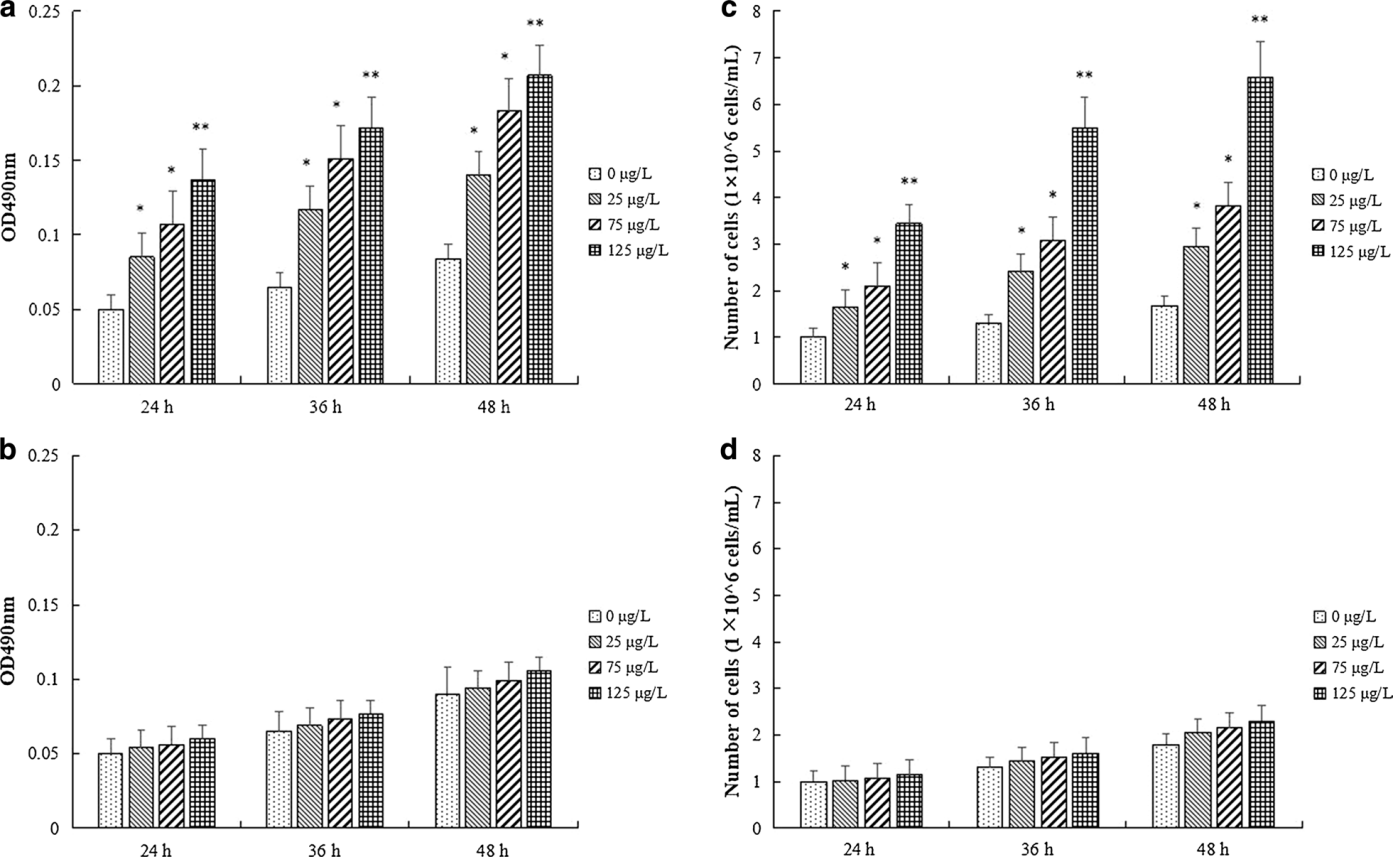
pGH protein biological activity. **a** Effect of different concentrations soluble pGH protein on proliferation of porcine intestinal epithelial cells by MTT assay. **b** Effect of different concentrations insoluble pGH protein on proliferation of porcine intestinal epithelial cells by MTT assay. **c** Effect of different concentrations soluble pGH protein on proliferation of porcine intestinal epithelial cells according to the number of dividing cells in culture. **d** Effect of different concentrations insoluble pGH protein on proliferation of porcine intestinal epithelial cells according to the number of dividing cells in culture. The results of three independent experiments were indicated as mean ± SD. *p < 0.05 indicated significant difference vs. 0 μg/L pGH treatment. **p < 0.01 indicated highly significant difference vs. 0 μg/L pGH treatment

The soluble pGH protein and insoluble pGH protein were used to stimulate cultured pig intestinal epithelial cells in vitro. The cell proliferation was measured by MTT method after 24 h, 36 h and 48 h, and the results were shown in Fig. 9. Compared with the control group, the soluble pGH protein in concentrations of 25 μg/L (*p *< 0.05), 75 μg/L (*p *< 0.05) and 125 μg/L (*p *< 0.01), had significant proliferative effects in pig small intestinal epithelial cells, while the insoluble pGH protein don’t. The stimulation index of soluble pGH protein was significantly superior to the observed for the insoluble protein (*p *< 0.05). For example, the relative proliferation rate of soluble pGH (125 μg/L) was 249% of the control group, while insoluble purified pGH protein (125 μg/L) was only 118% of the control group (*p *> 0.05). As expected, the trend of dividing cell number in culture was similar with the MTT data (Fig. 9c, d).

### Partial wall breakage of *Pichia pastoris* X33

**Fig. 10:**
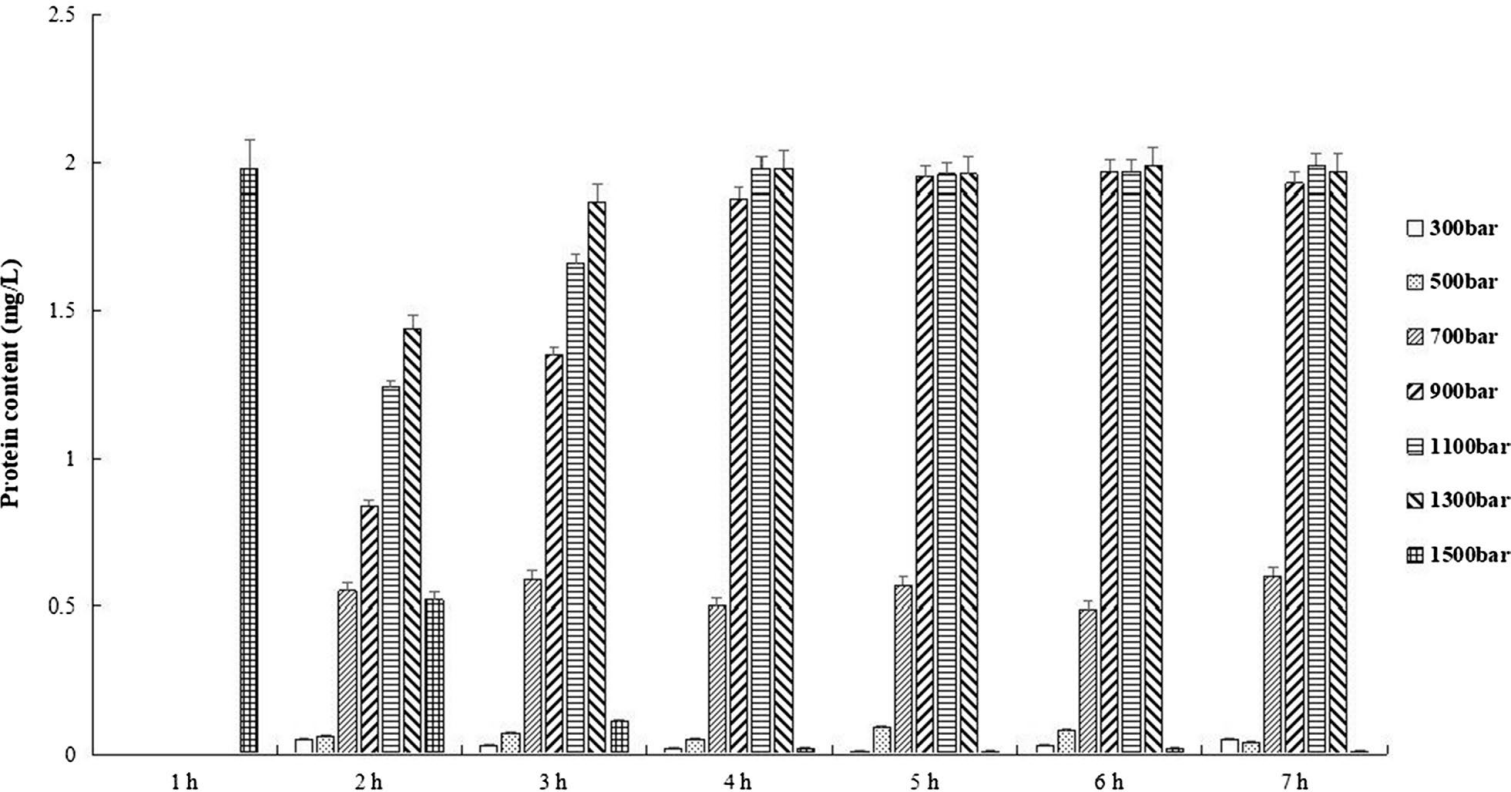
Effect of pressure parameters on cell wall breakage. The cell walls of *Pichia pastoris* were crushed at 300 to 1500 bars, respectively, and the content of soluble pGH protein was determined in 100 mL simulated intestinal fluid every hour (mg pGH protein per liter of simulated intestinal fluid). The results of three independent experiments were indicated as mean ± SD

The pGH1-Ssa1/Sis1 *Pichia pastori* X33 was crushed with 300, 500, 700, 900, 1100, 1300 and 1500 bar respectively, and the content of pGH after incubation in simulated stomach and intestinal fluid was measured (Fig. 10). The pGH1–Ssa1/Sis1 was fully released in 0 h with *Pichia pastoris* X33 crushed by 1500 bar. It is all known that the residence time of food in the intestine was about 4 h [37], and the data indicated that *Pichia pastoris* X33 crushed by 1300 bar released completely the pGH in the intestinal fluid at 3 h. Therefore, 1300 bar was selected to prepare partial broken-wall of *Pichia pastoris* X33 for further functional analysis.

### Clinical experimental analysis

**Fig. 11:**
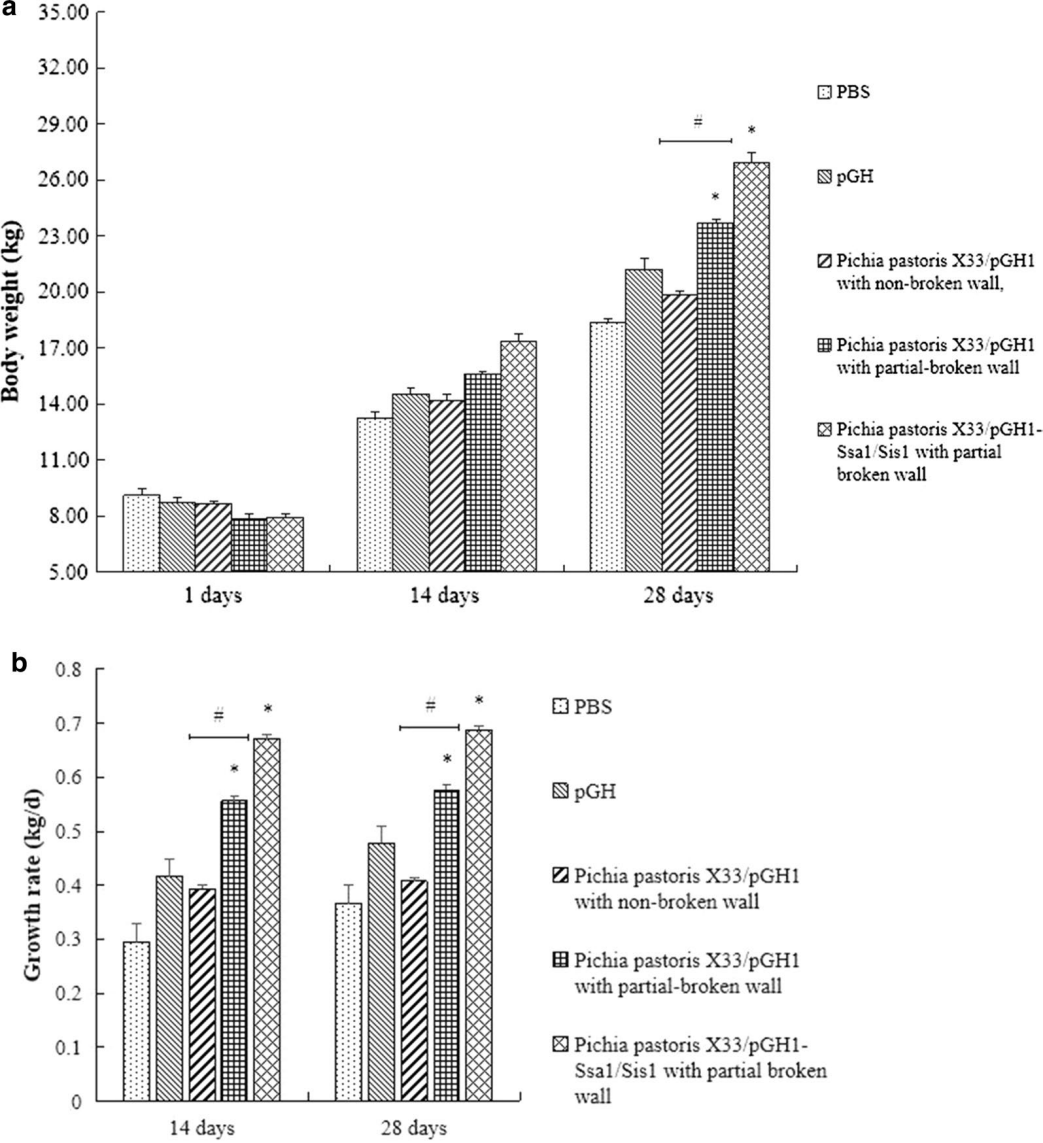
Animal experimental analysis. Piglets were orally administered with PBS, purified pGH, *Pichia pastoris* X33/pGH1 with non-broken wall, *Pichia pastoris* X33/pGH1 with partial-broken wall, and *Pichia pastoris* X33/pGH1–Ssa1/Sis1 with partial broken wall. The body weight was detected and growth rate was counted every 14 days. **a** The effect of pGH on the body weight of piglets. **b** The effect of pGH on growth rate of piglets. The results of three independent experiments were indicated as mean ± SD. *p < 0.05 indicated significant difference vs. PBS group, #*p *< 0.05 indicated significant difference between groups

Animal experiments were used to further validate the feasibility of our research. Animal experiment data showed that oral treatment with *Pichia pastoris* X33/pGH1 with partial-broken wall and *Pichia pastoris* X33/pGH1–Ssa1/Sis1 with partial broken wall had significant effects on the weight of piglets (*p *< 0.05 vs. PBS group) and the change of body weight and the growth rate was the most significant in *Pichia pastoris* X33/pGH1–Ssa1/Sis1 with partial broken wall treatment group (*p *< 0.05 vs. PBS group) (Fig. 11). Besides, animal experiments also confirmed that partial wall-breaking of *Pichia pastoris* was conducive to the release of intracellular substances and promoted the growth of piglets (*p *< 0.05 vs. *Pichia pastoris* X33/pGH1 with non-broken wall group). Compared to PBS group, the results further confirmed our assumption that co-expression of chaperone and partial wall-breaking could indeed promote effectively the biological activity of intracellular proteins.

## Discussion

pGH had a significant effect on muscle growth and nutrition distribution. However, the cost of extracting pGH from pituitary gland was too high for commercial applications in animal husbandry. Reports on the efficient expression of recombinant pGH protein by bacterial expression systems were still insufficient. Previous studies have focused on improving the yield of pGH by optimizing the conditions of extracellular secretion of pGH, while pGH intracellular soluble expression has rarely been reported [22, 39]. Moreover, the yield of recombinant pGH is not satisfactory in current expression systems, and pGH is easily degraded in vitro, which limits its biological activity. In this study, in order to improve the yield of pGH and its biological activity of pGH, the pGH gene was optimized and co-expressed with molecular chaperones from different sources and families to determine the optimal combination.

*Pichia pastoris* was developed as an efficient host for heterologous gene expression using promoter from the methanol-induce alcohol oxidase 1 (AOX1) gene [40], and can be grown to high cell-density. Moreover, *Pichia pastoris* is an excellent system for expressing heterologous proteins to high levels in soluble and secreted forms [41, 42]. Up to now, more than 1000 exogenous proteins have been successfully expressed in the *Pichia pastoris* expression system. The exogenous gene sequence was a key factor affecting expression level in *Pichia pastoris*. Synonymous codon usage bias is a common phenomenon among organism, and codon preference in genes have a significant effect on expression levels of the host [43, 44]. Codon optimization has been repeatedly demonstrated to significantly improve expression levels of exogenous functional genes [11, 45–48]. To improve expression level of pGH, pGH gene sequence was optimized according to the codon preference of *Pichia pastoris*. Both optimized and not optimized pGH genes could be successfully expressed in protein in *Pichia pastoris*, but after pGH gene optimization, the yield of recombinant pGH protein was significantly higher than the non optimized, which was consistent with the existing reports. However, there was no change in the yield of intracellular soluble pGH protein, which was still in low level. It was possible that pGH protein occurred in wrong fold, when it is overexpressed intracellularly in *Pichia pastoris*.

Molecular chaperones are proteins that have a role in facilitating the folding of the polypeptides as well as in their assembly into oligomeric structures, translocation and degradation [49]. In addition, molecular chaperones could effectively increase protein expression by co-expression [11]. In order to improve the intracellular soluble expression of pGH protein, *E. coli* chaperones GroEL–GroES, *Pichia pastoris* chaperones Ssa1–Sis1 and Bip–PDI were co-expressed respectively with pGH. Among the three molecular chaperones, the *Pichia pastoris* chaperone Ssa1–Sis1 resulted in the most significant increase in the yield of soluble pGH protein. Co-expression of molecular chaperones GroEL–GroES only slightly increased the expression level of soluble pGH protein. When the molecular chaperone Ssa1–Sis1 is co-expressed with pGH, the data showed that the total yield of pGH had not changed, while the intracellular solubility of pGH protein increased. When Bip–PDI was co-expressed with pGH, the insoluble pGH protein decreased, but the soluble pGH protein did not change, which was consistent with our previous findings [11]. It is reported that the processed proteins are selective in the process of GroEL–GroES assisting protein folding [50]. Kerner et al. used quantitative proteomics to analyze the entire *E.coli* proteome (about 2400 proteins), and found that there were about 250 proteins interacting with GroEL [51]. In those proteins, most of the proteins either used GroEL or used upstream molecules to achieve normal folding, and only about 85 proteins must rely on the GroEL–GroES system to fold normally, called a compulsive dependent substrate normally. In this study, pGH may not be a mandatory dependent substrate for the GroEL–GroES system, so the soluble expression is limited. The results of co-expression of *Pichia pastoris* chaperones and pGH indicated that the chaperone Ssa1–Sis1 could effectively promote protein correct folding and prevent aggregation. However, the chaperone Bip–PDI did not promote the correct folding of pGH protein, but could degrade the wrongly folded pGH.

To further increase the yield of pGH, the optimal temperature, pH and culture time for expression of pGH protein in *Pichia pastoris* were investigated by single factor experiments. Previous findings showed that low temperature induction resulted in more conducive to the expression of heterologous proteins in *Pichia pastoris* [52, 53]. In our study, the expression of pGH protein in *Pichia pastoris* was highest at 20 °C, pH 6.0, for 48 h, which mirrored the finding that mRNA is more stable at the low temperature [54].

So far, cell wall breaking was mostly used to release intracellular substances [27, 55]. In order to extract lipid from *Nannochloropsis* sp, cell walls were completely broken by combination of commercial enzymes [56]. Furthermore, ultrasonication together with enzymes were used to extract intracellular proteins from *Lactobacillus brevis 49* [57]. In most of related researches, the purpose of cell wall breaking was to extract intracellular substances. In this study, in order to achieve targeted release of pGH in intestine, we had partially broken the cell wall of *Pichia pastoris.* The experimental results show that partial wall breakage did achieve effective targeted release of pGH.

Animal experiments were used for final validation. The data illustrated that the technology of co-expression of molecular chaperone and partial wall-breaking could effectively promote the expression of biological activity of intracellular proteins. It is generally known that yeast is a high quality single-cell protein source, which is widely used in animal feeding, and also has better recombinant biological activity. Direct feeding of animals with pGH-rich yeast not only provide high quality protein for test animals, but also effectively protect the growth-promoting biological activity of pGH. By partially breaking the yeast wall, the cell wall is incomplete and easily broken in the animal’s intestinal tract, so that pGH can be released from the yeast cell in the intestinal tract in a targeted way, which suggest its possible application in pig industry. In this study, we had increased the intracellular soluble expression of pGH protein by optimizing codons and co-expression with molecular chaperones, and prolonged the half-life of pGH in vitro by partially breaking the wall. Besides, the targeted release of intracellular substances was achieved, and the degradation of pGH, a kind of substance that degrades easily in vitro, was effectively prevented in vitro, which is probably related to the further exertion of biological activity. Meanwhile, the subsequent tedious purification steps were avoided, which led to the reduction of cost.

## Conclusion

In this work, we optimized the pGH gene and screened three different chaperones co-expression systems with pGH. We found that gene optimization could effectively increase the yield of total pGH, and Ssa1–Sis1 chaperone significantly increased the expression of soluble pGH among the three chaperones. Due to its easy degradation in vitro, pGH needed to be embedded in liposomes in the breeding industry, which led to high-cost. In order to reduce the cost, in this study pGH was protected by partial cell wall instead of liposome embedding. It had been shown that partial cell wall breakage could effectively prevent the degradation of pGH in vitro, and achieved the targeted release of pGH. In addition, we obtained the production of intracellular soluble pGH protein up to 70 mg/L, insoluble pGH 270 mg/L and total pGH 340 mg/L by flask fermentation culture (at 20 °C, pH 6.0, for 48 h). To our knowledge, this is the first systematic study to increase the yield of pGH in *Pichia pastoris* and to promote its biological activity in vitro.

## Supplementary information

**Additional file 1: Fig. S1.** Substitution omega PCR for pGAPK(H)A construction. **Fig. S2.** Insertion omega PCR for pGAPKA-Ssa1-GPR construction. **Fig. S3.** Insertion omega PCR for pGAPKA-PDI-GPR construction. **Fig. S4.** The plasmids used in this study. **Fig. S5.** Standard curve of BSA standard solution. **Fig. S6.** PCR splicing results. **Fig. S7.** PCR splicing results. **Table S1.** Primers for PCR reactions. **Table S2.** The plasmids used in this study.

## Data Availability

All data generated or analysed during this study are included in this published article and its additional files.
