# Supplementary material for: Co-expressing GroEL–GroES, Ssa1–Sis1 and Bip–PDI chaperones for enhanced intracellular production and partial-wall breaking improved stability of porcine growth hormone
Source: Microb Cell Fact. 2020 Feb 18;19:35. doi: 10.1186/s12934-020-01304-5 (PMC7027120; doi:10.1186/s12934-020-01304-5)
Supplement: Supplementary file 1 — Additional file 1: Fig. S1. Substitution omega PCR for pGAPK(H)A construction. Fig. S2. Insertion omega PCR for pGAPKA-Ssa1-GPR construction. Fig. S3. Insertion omega PCR for pGAPKA-PDI-GPR construction. Fig. S4. The plasmids used in this study. Fig. S5. Standard curve of BSA standard solution. Fig. S6. PCR splicing results. Fig. S7. PCR splicing results. Table S1. Primers for PCR reactions. Table S2. The plasmids used in this study. [file 12934_2020_1304_MOESM1_ESM.docx]

**Co-expressing GroEL-GroES, Ssa1-Sis1 and Bip-PDI chaperones for enhanced intracellular production and partial-wall breaking improved stability of porcine growth hormone**

Jinbo Deng^1^$, Jiaoqing Li^1^$, Miaopeng Ma^1^, Peijing Zhao^1^, Feiping Ming^1^, Zhipeng Lu^1^ $, Juqing Shi^1^ $, Qin Fan^1^, Qianyi Liang^1^, Junhao Jia^1^, Jiayi Li^1^, Shuxia Zhang^1^, Linghua Zhang^1,2^*

1 Guangdong Provincial Key Laboratory of Protein Function and Regulation in Agricultural Organisms, College of Life Sciences, South China Agricultural University, Guangzhou, Guangdong 510642, China

2 Guangdong Laboratory for Lingnan Modern Agriculture, Guangzhou, Guangdong 510642, China

*Corresponding author. Tel.: 86-13650960875; Fax: 8620-85282180.

E-mail address: [lhzhang@scau.edu.cn](mailto:lhzhang@scau.edu.cn)

Address: Zhang LingHua

Microbiological staff room, College of Life Sciences,

South China Agricultural University,

Wushan Road, Tianhe district

Guangzhou

GuangDong

China

510642

E-mail address: [lhzhang@scau.edu.cn](mailto:lhzhang@scau.edu.cn)

$ These authors contributed equally to this work.

**Hyg-Zeo-R (Kan- Zeo-R)**

**Hyg-Zeo-F (Kan- Zeo-F)**

**Ins1**

**Ins2**

**Ins2**

**Ins1**

**pGAPZA**

**Zeo**

**pGAPZA**

**Hyg (Kan)**

**Hyg (Kan)**

**Hyg (Kan)**

**pGAPK(H)A**

Zeo: Zeocin resistance gene

Hyg: Hygromycin resistance gene

Kan: G418 resistance gene

**Starting**

**plasmid**

**Chimeric**

**primers**

**Omega-PCR**

**Substitution mode**

**plasmid**

**First PCR**

**products**

**plasmid**

**Final**

**plasmid**

Fig. S1 Substitution omega PCR for pGAPK(H)A construction


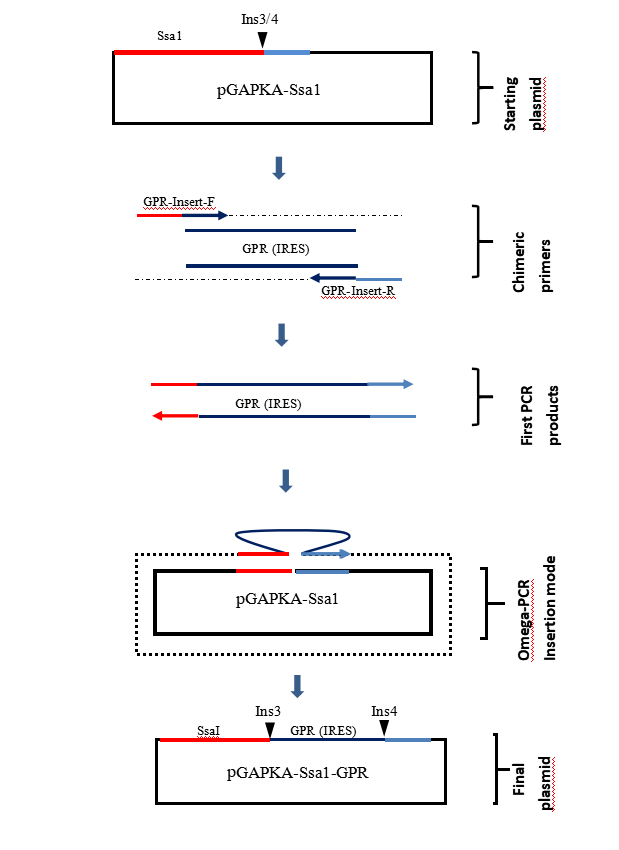


Fig. S2 Insertion omega PCR for pGAPKA-Ssa1-GPR construction


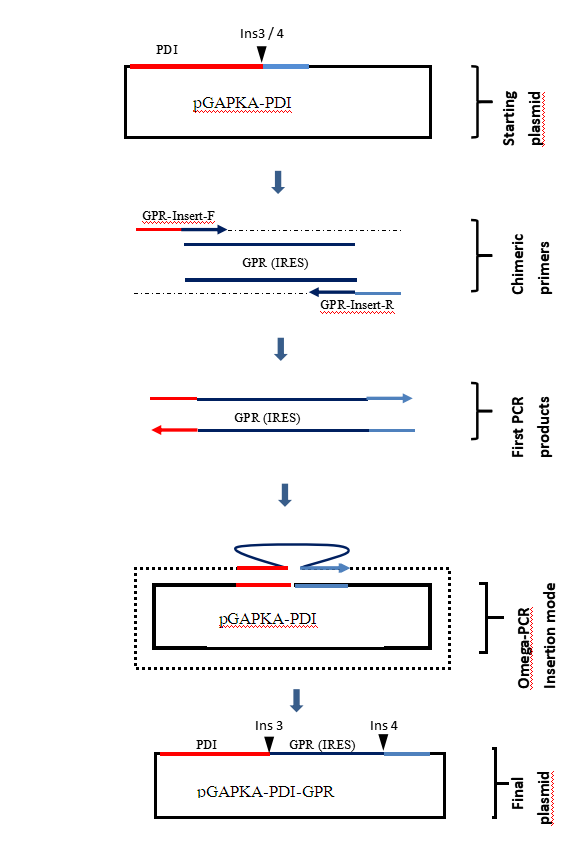


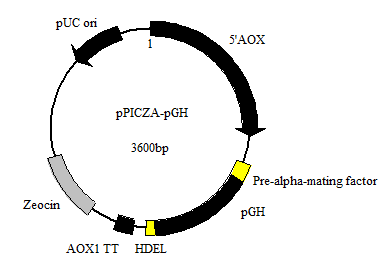

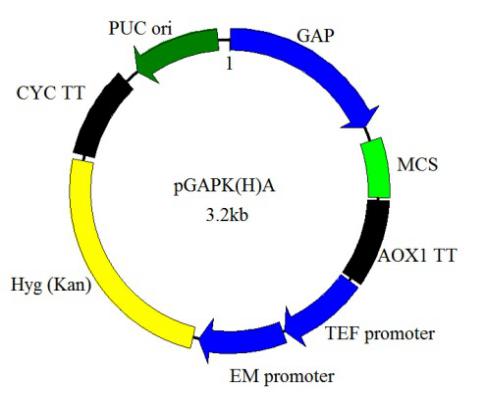

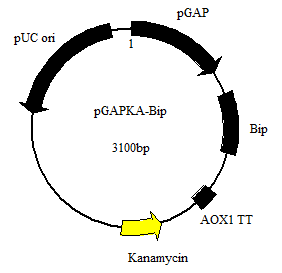

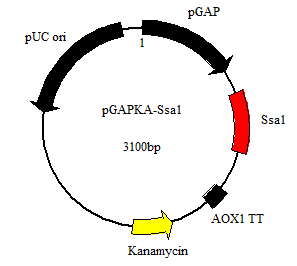


Fig. S3 Insertion omega PCR for pGAPKA-PDI-GPR construction


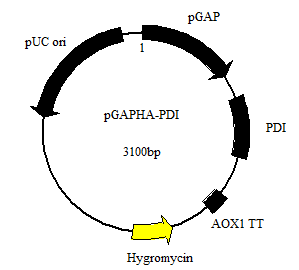

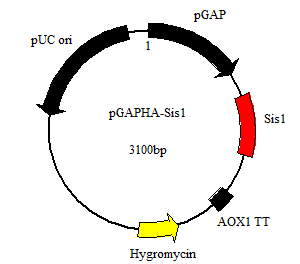


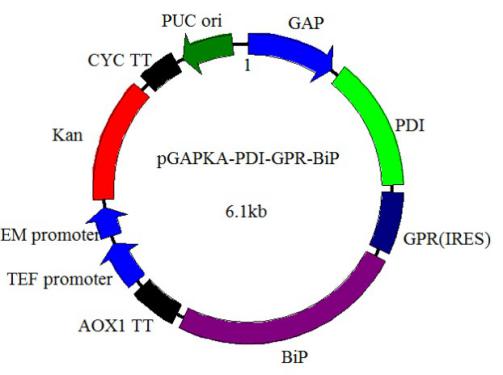

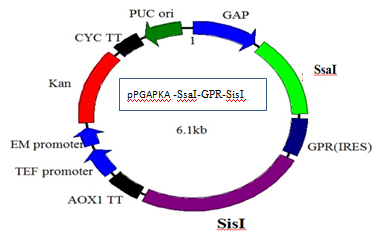


Fig. S4 The plasmids used in this study


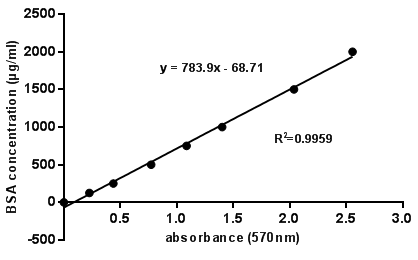


Fig. S5 Standard curve of BSA standard solution

**
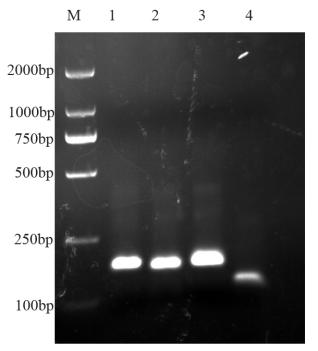
**

Fig. S6 PCR splicing results

Lane M was DL2000 standard molecular weight Marker, Lane 1, the result of the first group P1-P4 PCR splicing, Lane 2, the result of the second group P5-P8 PCR splicing, Lane 3, the result of the third group P9-P12 PCR splicing, and Lane 4, the result of the fourth group P13-P15 PCR splicing.


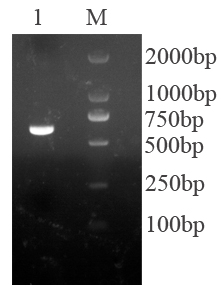


Fig. S7 PCR splicing results

Lane M, DL2000 standard molecular weight Marker, Lane 1, the result of PCR splicing of the target gene.

**Table S1 Primers for PCR reactions**

| Primer name | Sequencea |
| --- | --- |
| P1 | CCGGAATTCGCCACCATGTTTCCAGCTATGCCATTGTCTTCTTTGTTTGCTAACGCTG |
| P2 | GTATCAGCAGCCAATTGATGCAAATGTTGGGCTCTCAAGACAGCGTTAGCAAACAAAG |
| P3 | CAATTGGCTGCTGATACTTACAAGGAATTTGAAAGAGCTTACATTCCAGAAGGTCAAAG |
| P4 | CAGAGAAACAGAAAGCAGCTTGAGCGTTCTGAATAGAGTATCTTTGACCTTCTGGAATG |
| P5 | CAATTGGCTGCTGATACTTACAAGGAATTTGAAAGAGCTTACATTCCAGAAGGTCAAAG |
| P6 | CAACAAAGAGAATCTCAACAATTCAACATCAGATCTTTGTTGAGCTTCATCCTTACC |
| P7 | GTTGAGATTCTCTTTGTTGTTGATTCAATCTTGGTTGGGTCCAGTTCAATTCTTGTC |
| P8 | CAGAAGTACCAAAGACCAAAGAGTTAGTAAAGACTCTAGACAAGAATTGAACTGGAC |
| P9 | GGTCTTTGGTACTTCTGATAGAGTTTACGAGAAGTTGAAGGATTTGGAAGAAGGTATTC |
| P10 | GCTCTTGGAGAACCATCTTCCAATTCTCTCATCAAAGCTTGAATACCTTCTTCCAAATC |
| P11 | GATGGTTCTCCAAGAGCTGGTCAAATCTTGAAGCAAACTTACGATAAGTTTGATAC |
| P12 | CCGTAGTTCTTCAACAAAGCATCATCAGATCTCAAGTTAGTATCAAACTTATCGTAAG |
| P13 | CTTTGTTGAAGAACTACGGTTTGTTGTCTTGCTTCAAGAAGGATTTGCATAAGGCTG |
| P14 | GATTCAACAAATCTTCTACACTTCATAACTCTCAAGTAAGTTTCAGCCTTATGCAAATC |
| P15 | GTAGAAGATTTGTTGAATCTTCTTGTGCTTTCTAATAGCTCGAGCGG |
| PF | CCGGAATTCGCCACCATG |
| PR | CCGCTCGAGCTATTAGAAAG |
| GroEL-F | GGAATTCACCATGGCAGCTAAAGACGTAAAATTCGGTAACG |
| GroEL-R | ATAAGAATGCGGCCGCTTACATCATGCCGCCCATGCCACC |
| GroES-F | GGAATTCACCATGAATATTCGTCCATTGC |
| GroES-R | ATAAGAATGCGGCCGCTTACGCTTCAACAATTGCCAG |
| 5’AOX1 | GACTGGTTCCAATTGTTGACAAGC |
| 3’AOX1 | GCAAATGGCATTCTGACATCC |
| PGAP | GTCCCTATTTCAATCAATTGAA |
| Hsp70-F | ATGCCAGCTGTCGGTATTGATTTAGGAAC |
| Hsp70-R-Xba | GCTCTAGACTAATCGACTTCCTCAACAGTTGGTCCGT |
| Hsp70-F-Xho  Hsp40-F | CCGCTCGAGTTCAAACAAAATGCCAGCTGTCGGTATTGATTTAGGAAC  ATGGTGAAAGAACAAGGACTATACAAT |
| Hsp40-F-EcoR | CGGAATTCTTCAAACAAAATGGTGAAAGAACAAGGACTATACAAT |
| Hsp40-R-Not | CTTGCGGCCGCTTAAAACGCTTTGGAAATGGCATC |
| sig-F | ATGGCTGCTGGTCCAAGAACTTCAGTTTTATTGGCTTTCGCTTTGCTTTGTTTACCTTG |
| sig-R | AATGGCATTGCTGGAAACATAGCTCCAACTTCTTGAGTCCAAGGTAAACAAAGCAAAGC |
| sig-F/EcoR I | CGGAATTCTTCAAACAAAATGGCTGCTG |
| F-pre- alpha | ATGAGATTTCCTTCAATTTTTACTGCTGTTTTATTCGCAG |
| R-pre-alpha  R-HDEL-Not1 | TAATGGCATTGCTGGAAAAGCAGCTAATGCGGAGGATGCTGCGAATAAAACAGCAGT  CTTGCGGCCGCCTATTAAAGCTCGTCGTGGAAAGCGCAAGAGGATTCGAC |
| F-pre-alpha-EcoR1 | CGGAATTCTTCAAACAAAATGAGATTTCCTTCAAT |
| （wild）pgh-F | ATGTTGGGAGCCATGCCCTT |
| （wild）pgh-R | CTAGAAGGCACAGCTGCTC |
| phg-F-EcoRI | cggaattcaccATGTTGGGAGCCATGCCCTT |
| pgh-R-NotI | CTTGCGGCCGCCTAATGATGATGATGATGATGGAAGGCACAGCTGCTC |

**Table S2 The plasmids used in this study**

| Plasmids | Marker |
| --- | --- |
| pPICZA | Zeocin |
| pGAPZA | Zeocin |
| pPICZA-pGH（optimized）-HDEL | Zeocin |
| pPAO | Hygromycin |
| pPICZαA | Zeocin |
| pPICZA-pGH | Zeocin |
| pPICZαA-pGH | Zeocin |
| pPICZA-pGH（optimized ） | Zeocin |
| pGAPZA-GroEL-GroES | Zeocin |
| pGAPKA-Bip | Amp，Kan |
| pGAPHA-PDI | Hygromycin |
| pGAPKA-Ssa1 | Amp，Kan |
| pGAPHA-Sis1 | Hygromycin |

**Detailed procedure:**

**Native pGH cloing from cDNA:**

Full-length pGH gene splicing by overlapping PCR with optimized splicing primers P1-P15: using P1-P4 as the first group, P5-P8 as the second group, P9-P12 as the third group, and P13-P15 as the fourth group. These four groups were individually subjected to PCR with the following conditions:

20 μL reaction volume including 10 μL KOD-FX buffer, 0.2-0.4μL KOD-FX polymerase, 1~100 ng cDNA, 0.3 μM dNTP mix, 0.3 μM each forward and reverse primer. And the PCR cycling parameters were as follows: 94 ℃ for 2 min; 30 cycles of 98 ℃ for 10 s, 62 ℃ for 10 s, 72 ℃ for 15 s (1 kb/min) followed by a final 2 min extension.

**Optimized-pGH by splicing PCR:**

The four PCR products from above PCR were mixed, and primers PF/PR were used for the second round of splicing, the stitching was performed under condition as follows:

20 μL reaction volume including 10 μL KOD-FX buffer, 0.2-0.4μL KOD-FX polymerase, 1~100 ng cDNA, 0.3 μM dNTP mix, 0.3 μM each forward and reverse primer. And the PCR cycling parameters were as follows: 94 ℃ for 2 min; 30 cycles of 98 ℃ for 10 s, 53 ℃ for 10 s, 72 ℃ for 40 s (1 kb / min) followed by a final 2 min extension.

The results were shown in Fig. S6. The first to fourth groups of PCR products were added to the overlapping PCR reaction system, and splicing amplification was performed under splicing PCR conditions by primers PF/PR. The results were shown in Fig. S7.
